# Reduced mitochondrial fission and impaired energy metabolism in human primary skeletal muscle cells of Megaconial Congenital Muscular Dystrophy

**DOI:** 10.1038/s41598-021-97294-4

**Published:** 2021-09-13

**Authors:** Evrim Aksu-Menges, Cemil Can Eylem, Emirhan Nemutlu, Merve Gizer, Petek Korkusuz, Haluk Topaloglu, Beril Talim, Burcu Balci-Hayta

**Affiliations:** 1 Department of Medical Biology, Faculty of Medicine, Hacettepe University, 06100 Sihhiye, Ankara, Turkey; 2 Department of Analytical Chemistry, Faculty of Pharmacy, Hacettepe University, 06100 Sihhiye, Ankara, Turkey; 3 Department of Stem Cell Sciences, Graduate School of Health Sciences, Hacettepe University, 06100 Sihhiye, Ankara, Turkey; 4 Department of Histology and Embryology, Faculty of Medicine, Hacettepe University, 06100 Sihhiye, Ankara, Turkey; 5 Department of Pediatrics, Division of Child Neurology, Faculty of Medicine, Hacettepe University, 06100 Sihhiye, Ankara, Turkey; 6 Department of Pediatrics, Pathology Unit, Faculty of Medicine, Hacettepe University, 06100 Sihhiye, Ankara, Turkey; 7 Present Address: Department of Pediatrics, Yeditepe University, Istanbul, Turkey

**Keywords:** Cell biology, Molecular biology, Neuroscience, Diseases, Neurology

## Abstract

Megaconial Congenital Muscular Dystrophy (CMD) is a rare autosomal recessive disorder characterized by enlarged mitochondria located mainly at the periphery of muscle fibers and caused by mutations in the Choline Kinase Beta (*CHKB*) gene. Although the pathogenesis of this disease is not well understood, there is accumulating evidence for the presence of mitochondrial dysfunction. In this study, we aimed to investigate whether imbalanced mitochondrial dynamics affects mitochondrial function and bioenergetic efficiency in skeletal muscle cells of Megaconial CMD. Immunofluorescence, confocal and transmission electron microscopy studies revealed impaired mitochondrial network, morphology, and localization in primary skeletal muscle cells of Megaconial CMD. The organelle disruption was specific only to skeletal muscle cells grown in culture. The expression levels of mitochondrial fission proteins (DRP1, MFF, FIS1) were found to be decreased significantly in both primary skeletal muscle cells and tissue sections of Megaconial CMD by Western blotting and/or immunofluorescence analysis. The metabolomic and fluxomic analysis, which were performed in Megaconial CMD for the first time, revealed decreased levels of phosphonucleotides, Krebs cycle intermediates, ATP, and altered energy metabolism pathways. Our results indicate that reduced mitochondrial fission and altered mitochondrial energy metabolism contribute to mitochondrial dysmorphology and dysfunction in the pathogenesis of Megaconial CMD.

## Introduction

Megaconial Congenital Muscular Dystrophy (CMD) (OMIM 602541) is a rare form of congenital muscular dystrophy characterized by defective lipid biosynthesis. The disease has an estimated prevalence less than 1/1,000,000^1^. It is caused by loss-of-function mutations in *Choline kinase beta* (*CHKB*) gene encoding an enzyme that catalyzes the first step of phosphatidylcholine (PC) biosynthesis^2^. Clinically, Megaconial CMD is characterized by early onset muscle weakness, delay in motor development, severe intellectual disability without structural abnormalities in brain, autistic features and severe behavioral problems^2,3^. Dilated cardiomyopathy is observed in about 40% of the patients, while some patients present ichthyosis-like skin changes^3^. Although phosphatidylcholine is the major constituent in all biological membranes, mitochondria are known to be primarily affected in Megaconial CMD. The most characteristic histopathological feature of the disease is enlarged, megaconial mitochondria located mostly at the periphery of sarcoplasma, close to sarcolemma and depleted in the center of the skeletal muscle fibers^2–4^. Decreased respiratory chain complex activities were also observed by biochemical analysis and megaconial mitochondria with abnormal cristae were noted by electron microscopy in skeletal muscle biopsies of patients^5–8^. Functional studies of the *Chkb* knock-out mouse model, called as rostrocaudal muscular dystrophy (*rmd*) mice^9^ displayed impaired respiratory function, increased reactive oxygen species (ROS), and enhanced mitophagy in skeletal muscle tissue^10^. However, since this is a rare disease, there are no comprehensive studies to elucidate its pathogenesis in human biological samples.

Mitochondria are mobile organelles continuously undergoing fusion and fission events, collectively termed mitochondrial dynamics^11^. Together they serve as the most important quality control mechanism at the organelle level within the cell^12,13^. Mitochondrial fusion occurs in two steps by members of the dynamin-related GTPase protein family where the inner and outer membranes of two mitochondria fuse by GTP hydrolysis, in a coordinated manner. Mitofusin1 (MFN1) and mitofusin2 (MFN2), are anchored to the outer membrane and coordinate outer membrane fusion, while fusion of the inner mitochondrial membrane is accomplished by optic atrophy 1 protein (OPA1), which is tethered to the inner membrane facing the intermembrane space^14,15^. On the other hand, mitochondrial fission occurs in a single step in which dynamin-related protein 1 (DRP1) has the main role^16^. DRP1 is a dynamin family member GTPase found primarily in the cytosol and is recruited to the fission start sites which are associated with spotted vesicles attached to the endoplasmic reticulum and microtubules^17^. Two main receptor proteins, mitochondrial fission 1 protein (FIS1) and mitochondrial fission factor (MFF), cause DRP1 accumulation on the outer mitochondrial membrane and DRP1 squeezes both outer and inner membranes simultaneously by building up spirals around the organelle through its GTPase activity^18,19^. Mitochondrial morphology is largely dependent on the balance of fusion and fission events^20^, therefore extraordinary morphology of mitochondria suggests impaired balance of mitochondrial fusion and fission mechanisms in the pathogenesis of Megaconial CMD.

Although genetic background and the clinical features of the disease are known, no study has comprehensively investigated the effect of PC deficiency from the metabolomic perspective, which can give an understanding of the disease by revealing global metabolite changes in skeletal muscle^21,22^.

In this study, we hypothesized that imbalanced mitochondrial dynamics play a role in the pathogenesis of Megaconial CMD, and alter the mitochondrial bioenergetic efficiency of the skeletal muscle cells. We first investigated morphological characteristics of mitochondria, and the potential role of mitochondrial fusion and fission mechanisms in human primary skeletal muscle cells of Megaconial CMD patient. We next performed untargeted metabolomic profiling via Gas chromatography-mass spectrometry (GC–MS) and also identified phosphonucleotide levels of cells by targeted metabolomics via Liquid chromatography-mass spectrometry (LC–MS)/MS and then calculated the labeling ratios of the Krebs Cycle intermediates by fluxomic analysis. Here, we demonstrate for the first time that abnormal mitochondrial morphology in skeletal muscle of Megaconial CMD is associated with unbalanced mitochondrial dynamics and impaired mitochondrial energy metabolism, which was indicated by the observation of reduced mitochondrial fission and decreased levels of phosphonucleotide, Krebs cycle intermediates, and ATP.

## Results

### Defective mitochondrial network, morphology and localization in primary skeletal muscle cells of Megaconial CMD

**Figure 1:**
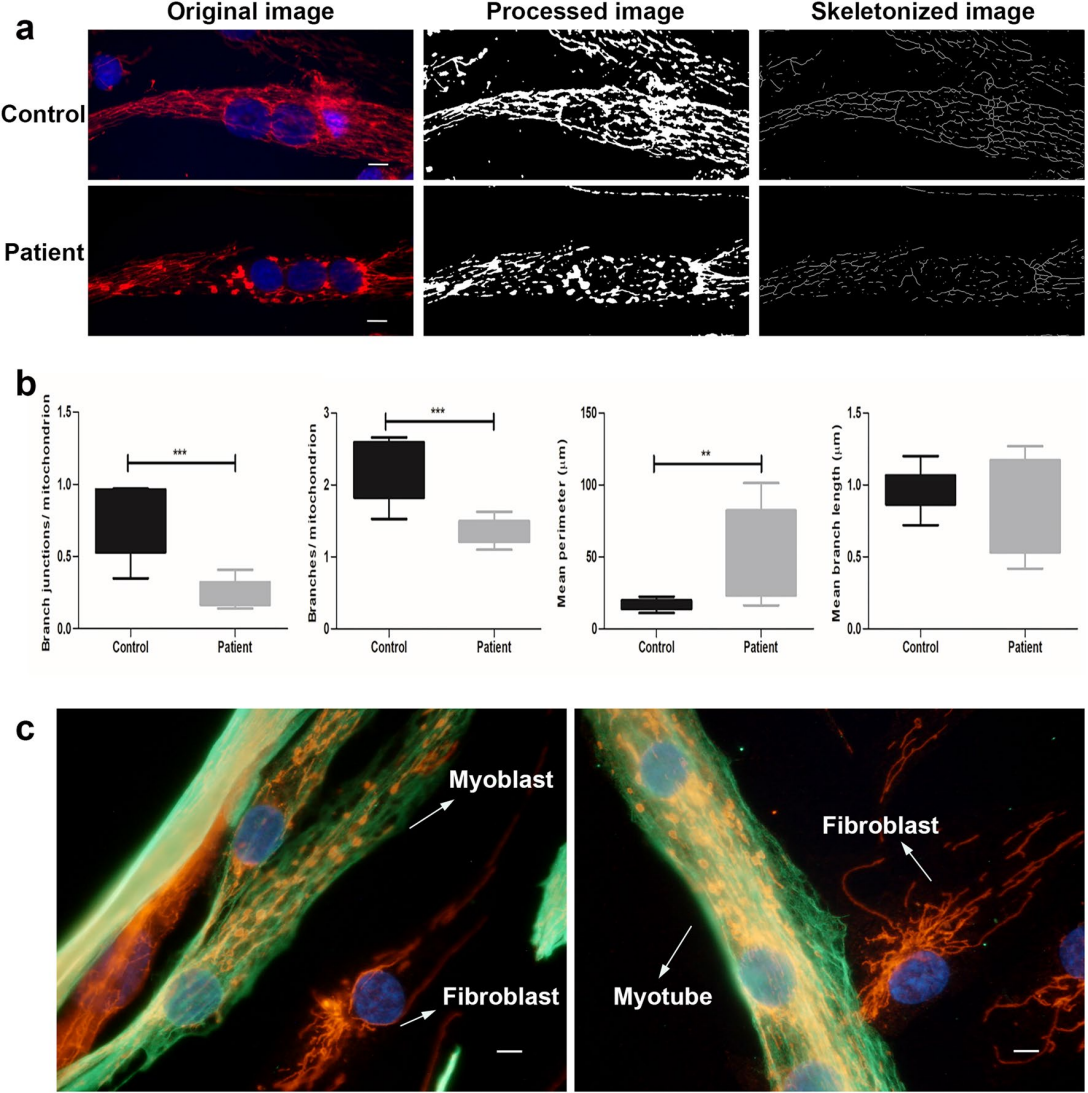
Mitochondrial morphology and network analysis in primary skeletal muscle cells of the Megaconial CMD patient (**a**) Immunofluorescence staining of the differentiated myotubes of the Megaconial CMD patient and the control by anti-TOM20 antibody (red), with nuclei counterstained with DAPI (blue). Original images were processed and skeletonized by ImageJ 1.53c software (NIH, https://imagej.nih.gov/ij/), as indicated. (**b**) Graphs showing the results of mitochondrial morphometric analysis. For all comparisons, differences between data sets were assessed by Mann–Whitney *U*-test with **p < 0.01, ***p < 0.001. (**c**) Images of mitochondrial morphology and network structure in myoblasts, myotubes and fibroblasts of the Megaconial CMD patient. Green/FITC: desmin/myoblasts and myotubes; Red/Texas Red: TOM20/mitochondria; blue: DAPI/nuclei. Scale bars: 10 μm. Analysis was performed on 50 primary skeletal muscle cells in both patient and control.

In order to analyze the mitochondrial network and morphology, differentiated myotubes were co-stained with antibodies against desmin, a marker of muscle cells, and TOM20, a mitochondrial translocase located in the outer membrane of the organelle. The mitochondrial network was preserved in the control myotubes and the mitochondria displayed elongated and filamentous morphology. Whereas the mitochondrial architecture of myotubes derived from the Megaconial CMD patient revealed some alterations including large and round (megaconial) mitochondria in a discontinuous network (Fig. 1a). Mitochondrial morphometric analysis by ImageJ indicated that the number of branches and branch junctions per mitochondrion were significantly decreased in patient cells comparing to that of the control, while mean branch length was similar in patient and control cells. In addition, it was observed that mean perimeter of mitochondria was significantly increased in patient cells when compared to control (Fig. 1b).

**Figure 2:**
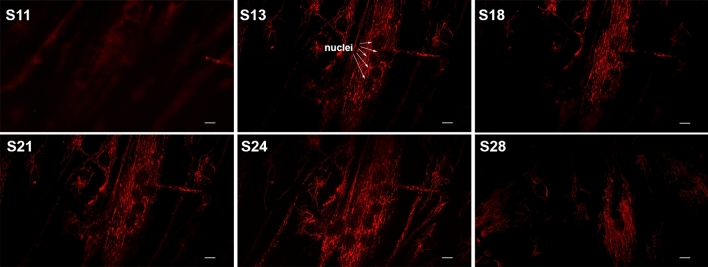
Representative confocal images of mitochondrial localization in differentiated myotubes of the Megaconial CMD patient. In the Z plane, 8-micron optical sections were taken from top to bottom and the optical section numbers are indicated on the figure. White arrows show the localization of the nuclei within the myotube. Red/Texas Red: TOM20/mitochondria, S: Section number. Scale bars: 10 μm.

It was observed that there was similar mitochondrial morphology in the Megaconial CMD myoblasts and myotubes, while organelle morphology and tubular network structure were preserved in the fibroblasts without desmin staining. It was shown that the damage in the organelle dynamics was detected only in muscle cells in primary cell culture (Fig. 1c). In addition, imaging of mitochondria by confocal microscopy revealed that megaconial mitochondria were concentrated close to the nucleus in the center of the differentiated myotubes of the patient (Fig. 2).

### Ultrastructural changes in primary skeletal muscle cells of Megaconial CMD

**Figure 3:**
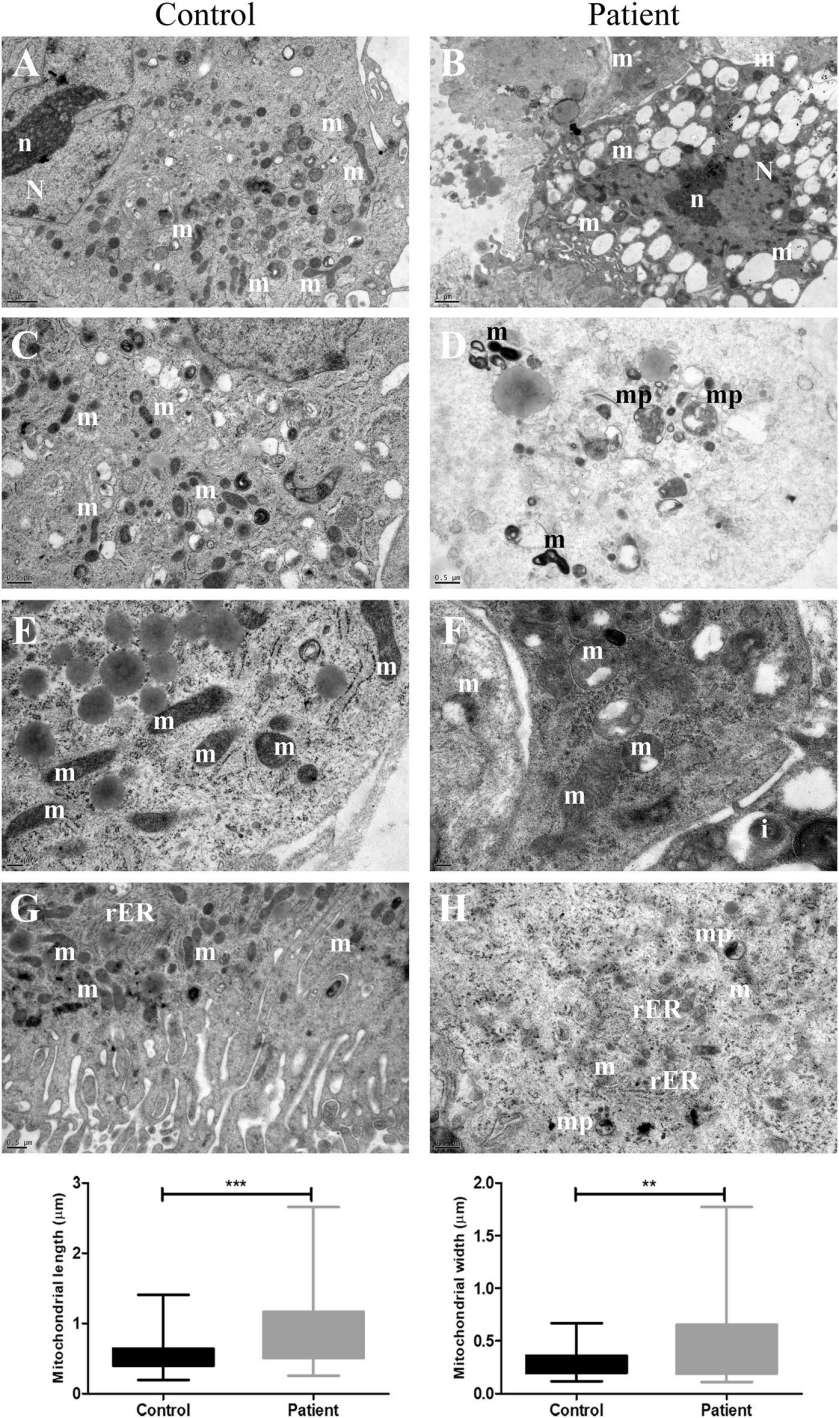
Transmission electron microscopy analysis of the differentiated Megaconial CMD myotubes. The electron micrographs of the control (**A**, **C**, **E**, **G**) and the patient (**B**, **D**, **F**, **H**). Note the abundant and pleomorphic, giant mitochondria in the patient’s sample. Mitochondria contain vacuoles and/or electron dense globular inclusions in (**D**, **F**, **H**). Giant, degenerated mitochondria present abnormal cristae and condensed structure in (**B**, **D**, **F**, **H**). Nucleus appears healthy in (**A**) and (**B**). (**G**) and (**H**) presents rough endoplasmic reticulum cisternae next to mitochondria. Uranyl acetate, lead citrate. N: Nucleus, n: Nucleolus, m: Mitochondria, i: Inclusion, mp: Mitophagic Vacuoles, rER: Rough Endoplasmic Reticulum. (**A**) 15,000×, (**B**) 12,000×, (**C**, **D**) 25,000×, (**E**, **F**) 40,000×, (**G**) 20,000×, (**H**) 25,000×. The graphs in the lower panel present median, min., max., values of the mitochondrial length and width for the patient and control myotubes. **p < 0.01, ***p < 0.001. Measurements were conducted using at least 50 mitochondria in both patient and control myotubes.

Both the control and the patient myotubes presented normal nuclear ultrastructure and well-preserved sarcolemma. The patient cells exhibited subsarcolemmal aggregates of pleomorphic mitochondria. Those mitochondria appeared enlarged, swollen, condensed and degenerated with their abnormal cristae. Different amplitudes of swelling, degeneration and mitophagy were noted for the mitochondria. The length (*p* < 0.001) and width (*p* < 0.01) of the patient’s mitochondria were significantly higher than those of the control (Fig. 3A–H). Some enlarged mitochondria presented vacuolization and/or dense globular inclusion-like structures. The inner and outer mitochondrial membrane generally remained intact. Mitochondria of the control cells appeared homogeneous in size and revealed healthy membranous structure although some cristae presented effacements as fixation artefacts (Fig. 3A–H). Rough endoplasmic reticuli appeared generally healthy with local dilatations at the neighborhood of mitochondria in both groups (Fig. 3G,H).

### Disruption of mitochondrial fission in primary skeletal muscle cells and skeletal muscle tissue of Megaconial CMD

**Figure 4:**
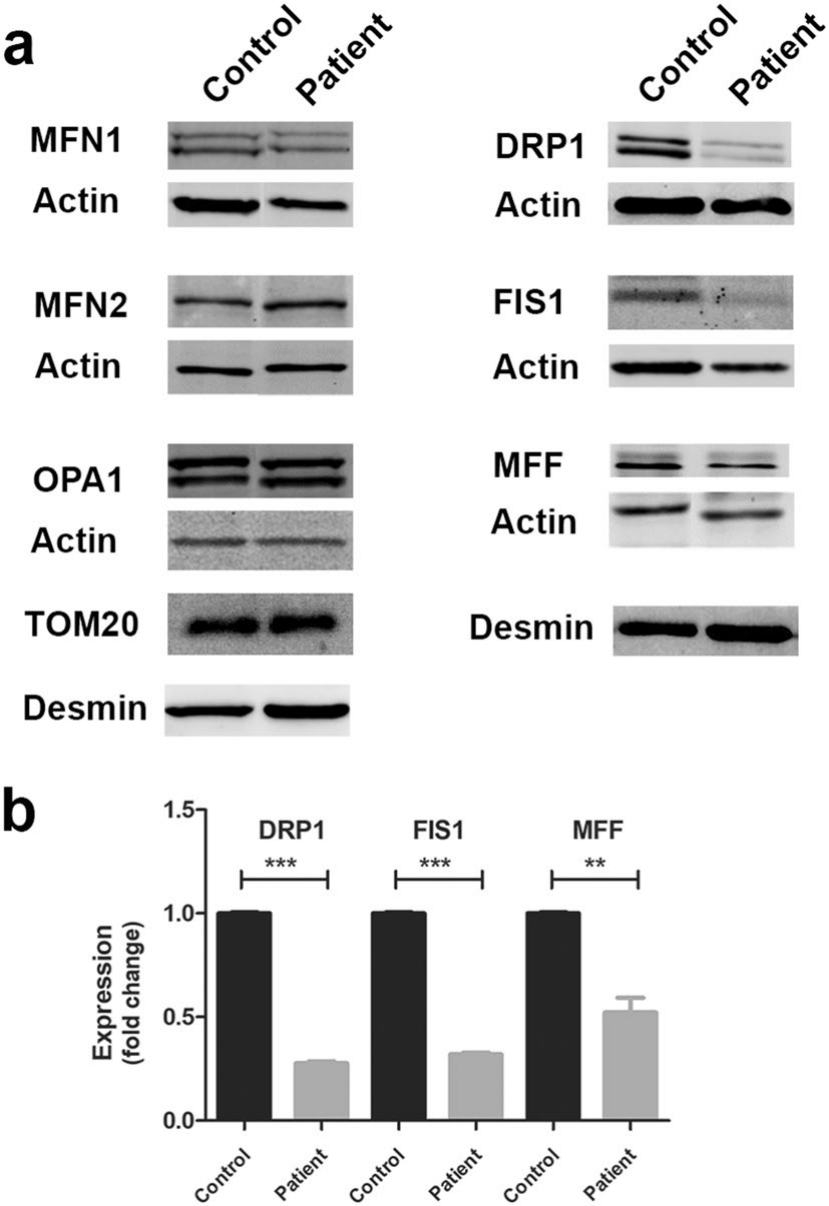
Western Blot and quantification analysis of proteins regulating mitochondrial dynamics in primary skeletal muscle cells of the Megaconial CMD patient. (**a**) Western blot images of proteins involved in mitochondrial fusion (MFN1: 84 kDa, MFN2: 86 kDa, OPA1: 80-100 kDa) and fission (DRP1: 79-84 kDa), MFF: 25-38 kDa), FIS1: 15 kDa). While actin was used to control equal loading, muscle specific expression patterns of proteins were eveluated by comparing the levels of proteins to desmin. (**b**) Graphs showing the quantitative analysis of Western blotting by ImageJ. The asterisk in the graph indicates the significant p value obtained by Student's *t-*test (**p < 0.01, ***p < 0.001), and the error bar indicates the standard deviation. Uncropped images of Western blotting are shown in Supplementary figure S1.

The expression levels of the basic proteins involved in mitochondrial fusion (MFN1, MFN2, OPA1) and fission (DRP1, MFF, FIS1) mechanisms were analyzed by Western blotting in differentiated myotubes of the Megaconial CMD patient and the control. As a result of Western blotting, no statistically significant difference was observed in the expression levels of mitochondrial fusion proteins, including MFN1, MFN2 and OPA1 in differentiated myotubes of the patient, compared to control cells. However, the expression levels of DRP1, FIS1, and MFF proteins involved in mitochondrial fission were significantly reduced in Megaconial CMD patient by 3.6, 3, and 2.1 times, respectively (Fig. 4).

**Figure 5:**
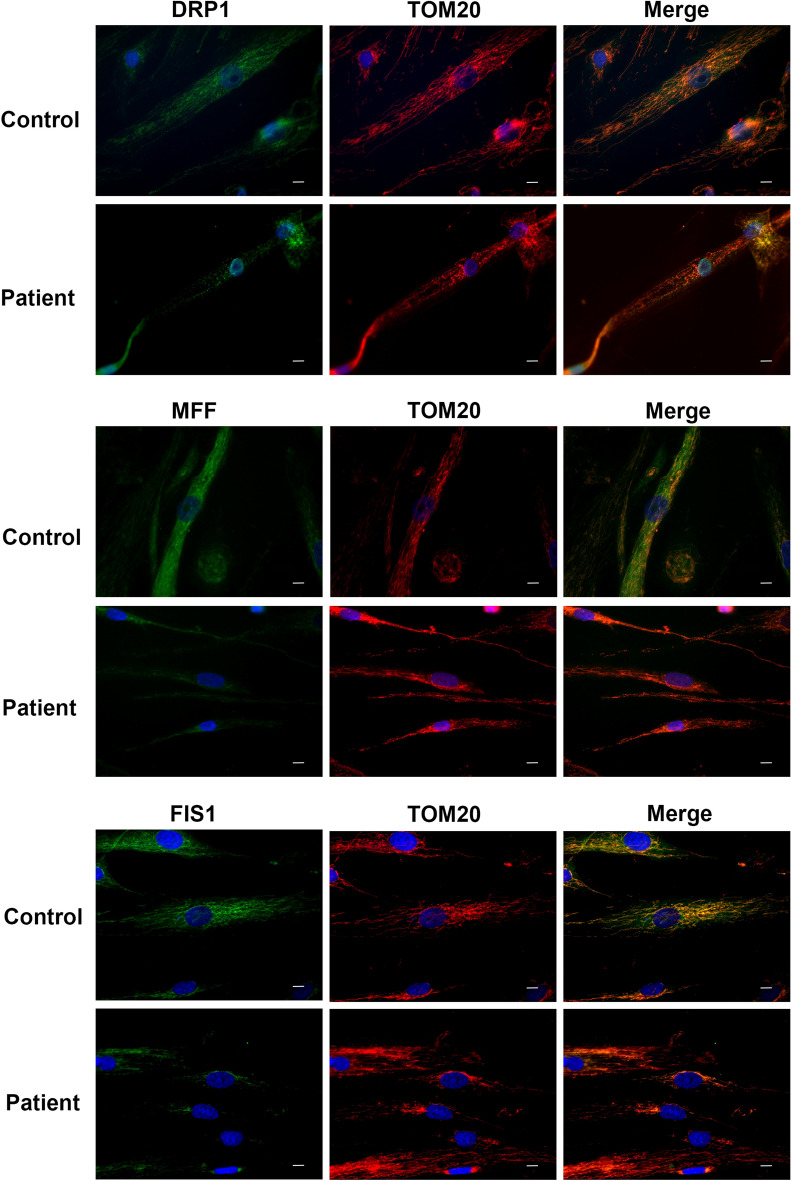
Immunofluorescence analysis of mitochondrial fission proteins in primary skeletal muscle cells of the Megaconial CMD patient. Left panel: Green/FITC: DRP1, MFF and FIS1 (top to bottom); Middle panel: Red/Texas Red: TOM20/mitochondria; Right panel: Merged images, Blue: DAPI/nuclei. Scale bars: 10 μm.

**Figure 6:**
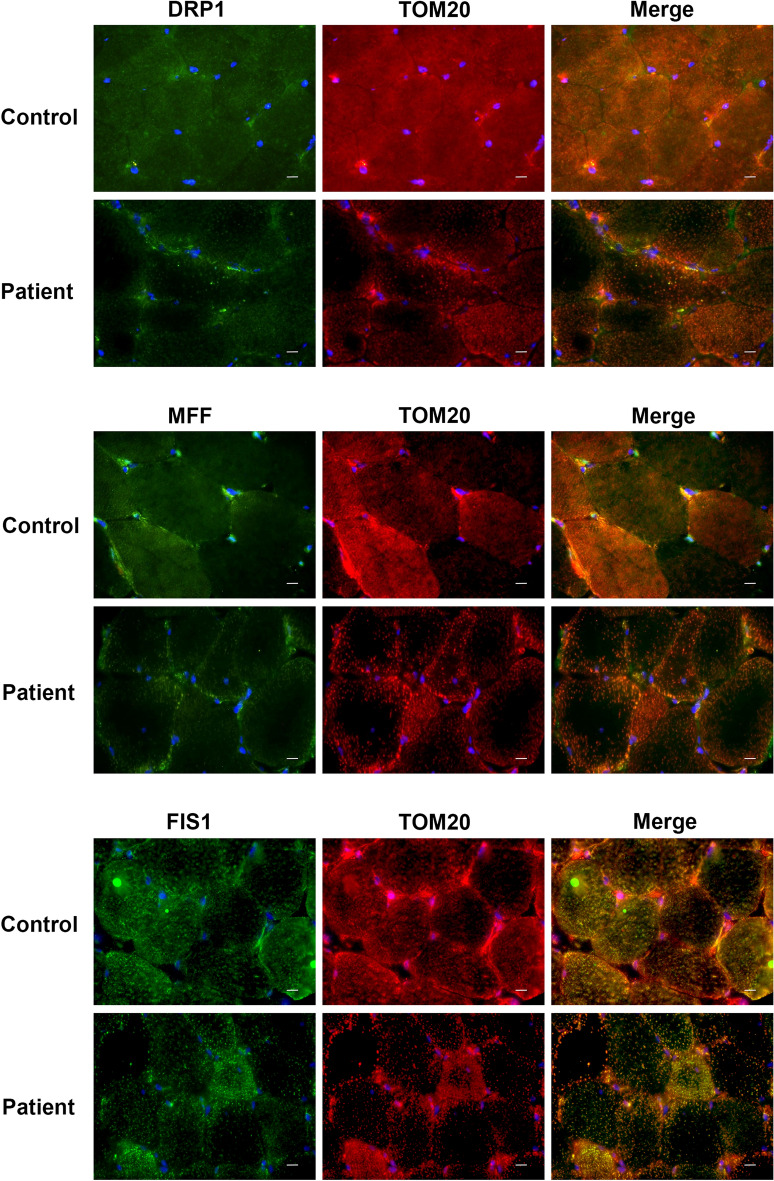
Immunofluorescence analysis of mitochondrial fission proteins in skeletal muscle tissue sections of the Megaconial CMD patient. Left panel: Green/FITC: DRP1, MFF and FIS1 (top to bottom); Middle panel: Red/Texas Red: TOM20/mitochondria; Right panel: Merged images, Blue: DAPI/nuclei. Scale bars: 10 μm.

Next, co-staining of TOM20 and three mitochondrial fission proteins (DRP1, MFF, FIS1) that showed decreased expression level by Western blotting was performed in primary skeletal muscle cells. A decrease in the expression level of all three proteins was also observed by immunofluorescence staining. In addition, while DRP1, FIS1 and MFF showed a homogeneous staining pattern in control cells, they were shown to be abundant in perinuclear region of the patient cells (Fig. 5). Immunofluorescence staining of transverse sections of skeletal muscle tissue indicated that TOM20, DRP1, MFF and FIS1 showed a more homogeneous staining pattern within control muscle fibers. However, in skeletal muscle sections of the Megaconial CMD patient, decreased staining of mitochondrial fission proteins was observed in addition to more granular mitochondria located mostly at the periphery of the fibers (Fig. 6).

**Figure 7:**
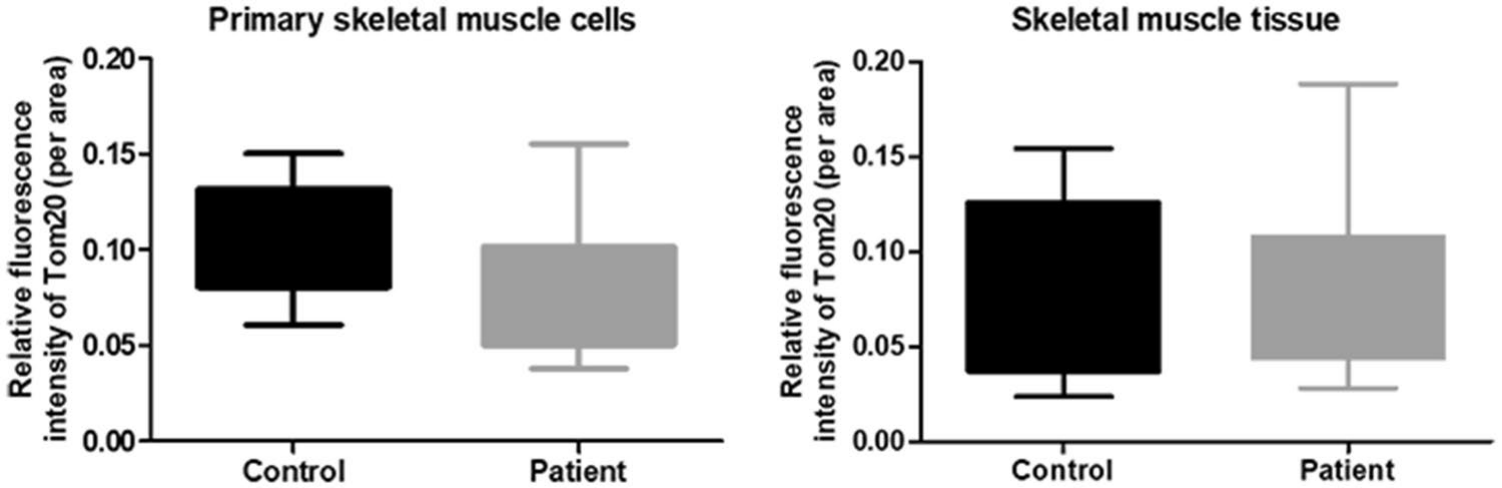
Fluorescence intensity analysis of TOM20 in primary skeletal muscle cells and skeletal muscle tissue sections of Megaconial CMD patient. Intensity quantifications were performed on 30 primary skeletal muscle cells and skeletal muscle tissue fibers in both patient and control samples.

To understand whether there is a difference in mitochondrial content between patient and control, fluorescence intensity analysis of TOM20 was carried out in primary skeletal muscle cells and also in skeletal muscle tissue sections. The results revealed that there is no statistically significant difference between patient and control samples (Fig. 7).

### Impaired mitochondrial energy metabolism in primary skeletal muscle cells of Megaconial CMD revealed by metabolomic and fluxomic analysis

**Figure 8:**
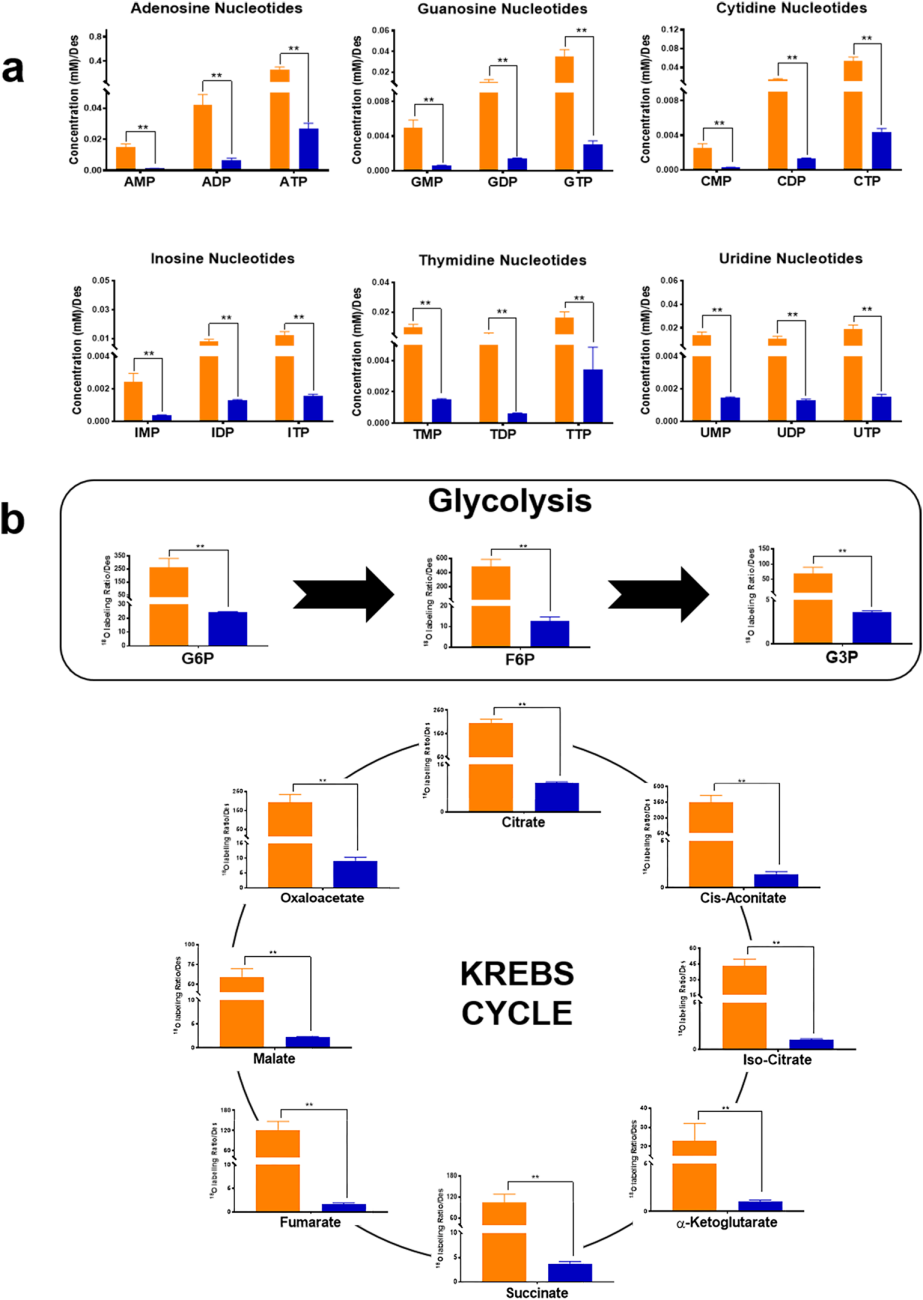
Metabolomic and fluxomic analysis in primary skeletal muscle cells of the Megaconial CMD patient. (**a**) The oligophosphonucleotide levels (mM/Desmin), (**b**) The ^18^O labeling ratios of Krebs cycle metabolites in Megaconial CMD and control, **p < 0.01.

Metabolomic and fluxomic analyses were conducted to explain the relationship between mitochondrial fission impairment and mitochondrial energy metabolism. After deconvolution and alignment of the chromatograms, 449 mass spectral features were detected. 72 of them were identified using Fiehn retention index library. The PLS-DA graphs showed that the primary skeletal muscle cells of the Megaconial CMD patient and the control individual effectively and distinctly separated into two groups and they presented different metabolomic profile (Supplementary Fig. S2A). As shown in Fig. 8a, targeted metabolomic analysis revealed that the amount of phosphonucleotides were significantly decreased in primary skeletal muscle cells of Megaconial CMD patient compared to control (*p* < 0.01). In addition, the mitochondria dynamics were evaluated using ^18^O labeling ratios of glucose-6-phosphate (G6P), fructose-6-phosphate (F6P) and glycerol-3-phosphate (G3P), citrate, cis-aconitate, isocitrate, alpha-ketoglutarate, succinate, fumarate, malate and oxaloacetate were calculated by fluxomic analyses, and the results showed that glycolysis and Krebs Cycle dynamics were significantly decreased in primary skeletal muscle cells of Megaconial CMD patient (Fig. 8b).

All data obtained from metabolomics, fluxomics and targeted oligophosphonucleotide analysis were found to be consistent. Pathway analysis revealed that aminoacyl-tRNA biosynthesis, pyrimidine metabolism, valine, leucine and isoleucine biosynthesis, Krebs cycle and alanine, aspartate and glutamate metabolism, glyoxylate and dicarboxylate metabolism, purine metabolism, and arginine biosynthesis were found to be significantly different (*p* < 0.05 and FDR < 0.05) between primary skeletal muscle cells of the Megaconial CMD patient and the control (Supplementary Fig. S2B).

## Discussion

Mitochondrial dysfunction underlies several muscular dystrophies, including Duchenne muscular dystrophy, and Collagen VI muscular dystrophies such as Ullrich congential muscular dystrophy^23–26^. Preservation of mitochondrial dynamics is essential for the morphology and the function of the organelle^27^. Megaconial CMD is a distinct form of CMD with multisystem involvement and extraordinary mitochondrial structural changes, due to a disruption of a PC metabolism^2,3^. To the best of our knowledge, there are less than 50 patients reported worldwide, however there may be unreported cases^2–6,8,28–38^. The biggest cohort of patients are reported from Turkey, including the original gene identification report^1,2^. Although several studies have been conducted using human skeletal muscle samples and mouse models to elucidate mitochondrial damage, the mechanisms underlying mitochondrial dysfunction in the pathogenesis of Megaconial CMD remains unclear. In this study, potential effect of mitochondrial dynamics on abnormal mitochondrial phenotype in the pathogenesis of Megaconial CMD was investigated by using primary skeletal muscle cells and skeletal muscle biopsy and it was found that mitochondrial fission and fusion balance is impaired, and mitochondria are less prone to fission in primary skeletal muscle cells. In Western Blot experiments, a statistically significant decrease was observed in the expression levels of mitochondrial fission proteins DRP1, MFF and FIS1 in patient myotubes, while no statistically significant difference was found in the expression levels of mitochondrial fusion proteins between control and the patient. This decrease in the expression levels of fission proteins has also been confirmed by immunofluorescence analysis in primary skeletal muscle cells and skeletal muscle sections.

Distinct groups have suggested that DRP1 downregulation may contribute to swollen mitochondrial morphology. For instance, Lee et al. recognized huge, balloon-like mitochondria, similar to megaconial mitochondria seen in the pathogenesis of Megaconial CMD, in DRP1 knock-down HeLa cells. However, they did not observe such a mitochondrial morphology when FIS1 was knocked down^39^. In another study, Kageyama et al. showed that mitochondria were swollen and respiration was impaired in DRP1 knock-out mouse neurons^40^. Similar abnormal mitochondrial morphology was detected in fibroblasts of Parkinson’s patient carrying PTEN-induced kinase 1 (PINK1) mutation and this morphology has detected to be associated with a decrease in DRP1 expression levels^41,42^. It has been shown that knock-down of DRP1 adaptor proteins, MFF and FIS1, reduces mitochondrial fission in mouse embryonic stem cells^18^. Additionally, it has been proposed that inhibition of mitochondrial fission prevents cytochrome C release and downregulates apoptosis^39,43^. Therefore, the reason for downregulation of DRP1, FIS1, and MFF may be to protect cells from apoptosis.

Primary myoblast cell cultures are heterogeneous cultures that coexist with fibroblasts, and even when myogenic differentiation is induced in the culture medium, some myoblasts may also be present in the cell culture, since not all cells differentiate into myotubes synchronously. The specific megaconial mitochondria phenotype detected in the skeletal muscle tissue of Megaconial CMD patients in previous studies^2,4^ was also observed in primary myoblast cells and differentiated myotubes in our study. Interestingly, it was observed that morphological organelle damage was detected only in muscle cells and was not detected in fibroblasts that coexist with skeletal muscle cells found in primary culture. However, in terms of proliferation state, cells in the primary culture may not have the same cellular and organelle morphology due to the asynchronous cell cycle^44,45^. Although the mitochondrial network was seen to be disrupted in all Megaconial CMD myoblasts and myotubes, the number of swollen mitochondria may differ. Similarly, mitochondrial morphology shows heterogeneity even in different fiber types in skeletal muscle tissue^4,46^. In addition, mitochondria are typically localized at the periphery of the skeletal muscle fibers in Megaconial CMD^4^. However, in our study it was shown that mitochondria of both patient myoblasts and myotubes were located mostly close to the nuclei, away from the periphery of the cell. Mitsuhashi and Nishino have shown an increase in PINK1 and Parkin expression levels as mitophagy markers in skeletal muscle biopsies of Megaconial CMD patients^10^. In another study, overexpression of PINK1 and Parkin was found to alter the cellular localization of mitochondria, leading to accumulation around the nuclei^47^. For this reason, mitochondrial localization, which was mostly detected around the nuclei of Megaconial CMD patient derived cells, may be a result of increased mitophagy. The presence of mitophagy was also shown by ultrastructural examination in this study.

It has been suggested that the main PC biosynthesis pathway, known as the Kennedy pathway, becomes dysfunctional due to *CHKB* gene mutations, and this may activate the mitochondria-associated Endoplasmic reticulum (ER) membrane (MAM) pathway, which is the alternative PC synthesis pathway^6^. Although no significant morphological change was detected in the ER, we observed increased co-localization of ER and mitochondria in primary skeletal muscle cells of the patient (Supplementary Fig. S3). Moreover, alteration of the phospholipid content of the mitochondria can lead to impaired lipid-protein interactions^48,49^. Therefore, the reduction of PC level in Megaconial CMD mitochondrial membranes may have altered the lipid-protein interaction and this may have caused a decrease in the level of mitochondrial fission.

There are not any studies of Megaconial CMD in the context of metabolomic or fluxomic analysis. Since Megaconial CMD is characterized by enlarged mitochondria, we performed mitochondria-targeted analysis including detection of phosphonucleotide pools of the cell and the mitochondrial energy dynamics using stable isotope labeling. Within the scope of this study, we discovered for the first time that many metabolic pathways were found to be associated with mitochondrial dysfunction. Particularly, the production rate of ATP and Krebs cycle intermediates were decreased in primary skeletal muscle cells of the Megaconial CMD patient. Moreover, the low labeling ratio of G6P and F6P in primary skeletal muscle cells of the Megaconial CMD patient revealed a lower glycolysis rate. Additionally, the G3P labeling ratio indicated deficient activity of G3P shuttle and substrate supply to mitochondria^50^. All these results showed a deficiency in cellular bioenergetics by linking cytosolic metabolic networks to mitochondrial oxidation reactions^50^.

A cell must produce nucleotides, fatty acids and energy in order to maintain cellular homeostasis and these three metabolic pathways are linked together in the mitochondria^51^. We observed that Krebs cycle, which plays a leading role in energy production, and purine and pyrimidine metabolism were significantly downregulated in Megaconial CMD. Altered tRNA biosynthesis detected in our study has been reported as an indicator of mitochondrial dysfunction in mitochondrial diseases^52,53^. Moreover, the biosynthesis of valine, leucine, and isoleucine pathway, related to mitochondrial biogenesis^54^, and the glyoxylate and dicarboxylate metabolism, a shortcut for the Krebs cycle, and alanine, aspartate and glutamate metabolism, major entry points to the Krebs cycle, were significantly altered in our study. All these pathways indicate a disruption of cellular redox potentials due to improper mitochondrial function^55^.

In consistent with our results, decreased activities of respiratory chain complexes were reported in skeletal muscle tissues of some Megaconial CMD patients^5,8,34^. Moreover, in a study of *rmd* mice, decreased number of mitochondria due to increased mitophagy was shown to cause decrease in energy production^10^. Increased volume of mitochondria is known to induce higher ATP production due to increased total crista surface area^56^. Interestingly, although our TEM-based ultrastructural analysis showed that the mitochondria of the Megaconial CMD myotubes were enlarged, our -omics studies revealed a decrease in the ATP level. In addition to TEM-based analysis, our mitochondrial morphometric analysis which reveals degree of branching of the mitochondria, and the length of these branches showed a decrease in the number of branches and branch junctions per mitochondrion in the skeletal muscle cells of the patient. This result suggests that reduced ATP production may be due to the disruption of the mitochondrial network and the transformation of mitochondria into large spherical, individual (discrete) organelles in skeletal muscle cells of the patient. Although it may seem that the mitochondrial content was reduced in the primary skeletal muscle cells and tissue sections of the patient in some fluorescence microscopy images, fluorescence intensity analysis of TOM20 revealed no statistically significant difference between the patient and control cells and muscle fibers. As mitochondria of the patients are larger than controls, disruption of mitochondrial network and branching detected in patient cells and tissues may not affect total mitochondrial content compared to controls. In addition, reduced expression levels of DRP1, FIS1 and MFF are not due to decreased mitochondrial content, as no statistically significant difference in the expression levels of mitochondrial fusion proteins, MFN1, MFN2 and OPA1, was observed in differentiated myotubes of the patient, compared to control cells. All these results suggest a deficiency in cellular bioenergetics by linking cytosolic metabolic networks to mitochondrial oxidations^50^. However, it is difficult to know whether these metabolic changes are the cause or consequence of the disease.

The major limitation of this study is the sample size. Primary skeletal muscle cells and skeletal muscle biopsy were investigated from one patient, each. Therefore, the study should be performed with an increased number of patients to increase the accuracy of the results. Because Megaconial CMD is a rare disease, it may be difficult to access biological samples of newly diagnosed patients. Also, there are no other primary skeletal muscle cell lines in EuroBioBank, a unique network of rare disesase biobanks.

Our novel findings, namely impaired mitochondrial fission and energy metabolism in primary skeletal muscle cells derived from Megaconial CMD patient, represent an important advancement in understanding the mechanisms underlying mitochondrial dysfunction associated to disease pathogenesis.

## Materials and methods

### Primary skeletal muscle cells and culture conditions

All experimental protocols in this study were approved by the Hacettepe University Faculty of Medicine Ethical Review Board (GO 15/421-31), and all experiments were performed in accordance with relevant guidelines and regulations. Primary skeletal muscle cells of a Megaconial CMD patient (sample ID: NH10-1412A) harboring a homozygous mutation of c.722A>G (NM_005198.3) in the *CHKB* gene were supplied by the MRC Centre for Neuromuscular Disease Biobank London (REC reference number 06/Q0406/33). Primary skeletal muscle cells derived from a muscle biopsy with no diagnostic pathology as indicated by normal histomorphology was used as a control. Cells were expanded in Skeletal Muscle Cell Growth Medium (PromoCell) supplemented with 2 mM L-glutamine (Biowest) and 1% Pen/Strep (Biowest). Upon reaching 70–80% confluence, the medium was changed to a differentiation medium based on the Skeletal Muscle Cell Differentiation Medium (PromoCell) supplemented with 1% Pen/Strep (Biowest).

### Human skeletal muscle biopsies

During validation analysis with immunofluorescence staining, we examined skeletal muscle tissue sections of a Megaconial CMD patient carrying a homozygous mutation of c.922C>T (NM_005198.4) in the *CHKB* gene, and a control individual in whom a neuromuscular disease was excluded by both clinical and histopathological criteria. Written informed consent was obtained from all patients at the time of diagnostic muscle biopsy. All patients have been investigated in the Department of Pediatrics at Hacettepe University Faculty of Medicine and open biopsies were taken from vastus lateralis muscle at the time of diagnosis. The muscle biopsy specimens were rapidly frozen in isopentane cooled in liquid nitrogen and kept at − 80 °C until use. Serial transverse muscle sections were cut by cryostat for immunofluorescence staining.

### Antibodies

The primary antibodies, and their dilutions were as follows: anti-MFN1 (WB: 1/1000, Abcam, ab57602), anti-MFN2 (WB: 1/1500, Abcam, ab56889), anti-OPA1 (WB: 1/1000, BD Biosciences, 612606), anti-FIS1 (WB: 1/1000, IF:1/100, Novus Biologicals, H00051024-M01), anti-DRP1 (WB: 1/500; IF: 1/100, BD Biosciences, 611112), anti-MFF (WB: 1/500, IF:1/100, Santa Cruz Biotechnology, sc-398617), anti-TOM20 (WB:1/1000, IF: 1/100, Santa Cruz Biotechnology, sc-11415), anti-calnexin (IF: 1/200, Santa Cruz Biotechnology, sc23954), anti-PDI (IF:1/100, Abcam, ab2792), anti-desmin (WB: 1/200, IF: 1/50, Sigma-Aldrich, D8281) and anti-actin (WB: 1/1000; Sigma-Aldrich, G8795). HRP-conjugated goat anti-mouse and goat anti-rabbit (1/5000, Thermo Scientific) secondary antibodies were used for western blotting, while Alexa Fluor 488 and 568 conjugated secondary antibodies (1/500, Thermo Scientific) were used for immunofluorescent labeling.

### Protein isolation and Western blotting

Primary skeletal muscle cells were differentiated into multi-nucleated myotubes for 14 days and detached from the culture flasks with trypsin–EDTA (Biowest). After centrifugation at 2000 rpm for 10 min at room temperature, pellet was resuspended with protein isolation buffer containing 10 mM Trizma-base, 300 mM NaCl, 2 mM EDTA, 0.5% Triton-X-100 and Protease Inhibitor Cocktail (Roche). Then, cells were sonicated on ice for 20 secs for 9 times at 50% amplitude. After sonication, cells were centrifuged at 14,000 rpm for 10 min at + 4 °C and supernatant was collected. Total protein concentrations were determined using Pierce BCA Protein Assay Kit (Thermo Fisher Scientific, Waltham, MA, USA) following the manufacturer’s instructions. Equal amounts of proteins (30 μg) were run on 12% polyacrylamide gel and transferred to a polyvinylidene difluoride (PVDF) membrane (Immobilon-P; Millipore, Billerica, MA) via semi-dry transfer that was performed at 25 V for 30 min. Proteins were labelled with specific primary antibodies overnight at + 4 °C, followed by appropriate HRP-conjugated secondary antibodies for 1 h at room temperature. Blots were detected by SuperSignal West Femto Maximum Sensitivity Substrate (Thermo Scientific) with GeneGnome5-Chemiluminescence imaging system (Syngene) and the intensity of bands was quantified by using ImageJ 1.53c software (NIH, https://imagej.nih.gov/ij/). GAPDH was used to check equal protein loading of each lane. In order to analyze muscle specific expression patterns of proteins, all results were normalized according to desmin ratios.

### Immunofluorescence staining

Both skeletal muscle tissue cryosections (7 μm thick) and primary skeletal muscle cells differentiated into multi-nucleated myotubes for 14 days on glass coverslips were fixed with 4% PFA. After washing with 1xPBS, cells and tissue sections were permeabilized with 0.2% Triton X-100 for 10 min and then blocked with 1% BSA and 10% goat serum in 0.1% Tween 20 for 1 h at room temperature. Cells and tissue sections were immunostained by primary antibodies overnight at + 4 °C. After washing with 1xPBS, they were labelled with appropriate secondary antibodies for 1 h at room temperature. Immunostained cells and tissue sections were mounted with Prolong gold antifade mountant (Thermo Fisher) and observed under a fluorescent microscope (Carl-Zeiss Axioplan 2) and/or a confocal microscope (Leica DMI 4000 with Andor DSD2 Spinning Disk Attachment). Appropriate excitation and barrier filters were used to observe fluorescence.

The mitochondrial network and morphology were evaluated by ImageJ^57^. Images were first pre-processed and thresholded, next post-processed and particles were analyzed in terms of perimeter of mitochondria by Analyze Particles plugin of ImageJ. Processed images were then skeletonized and the mitochondrial network was evaluated in terms of number of branches, number of branch junctions and mean branch length by using the Analyze Skeleton 2D/3D plugin of ImageJ. At least 40 cells per culture were analyzed for both patient and control cells, and the data were collected from the average statistical value per condition. Also, fluorescence intensity analysis of TOM20 was carried out in ImageJ. 30 primary skeletal muscle cells/ skeletal muscle tissue fibers in both patient and control samples were quantified in terms of fluorescence intensity.

### Transmission electron microscopy (TEM)

Differentiated myotubes of the patient and the control were removed from the culture flask by a scraper and transferred to a conical tube. The medium was removed and cells were fixed with 2% glutaraldehyde solution in PBS at room temperature for 30 min. The samples were post-fixed in 1% osmium tetroxide at room temperature in dark for 1 h, embedded in 2% agar, dehydrated in a series of ethanol, cleared in propylene oxide and embedded in epon plastic blocks (Agar Scientific, UK)^58^. The ultra-thin sections were stained with uranyl acetate and lead citrate, analyzed with a TEM and attached digital camera (TEM, JEM1400, Japan, GATAN, Germany). The length and width of mitochondria (at least 50 mitochondria per section) were measured with DigitalMicrograph (Gatan Inc., USA) software^6,59,60^.

### Untargeted metabolomic analysis

The cell extracts of the Megaconial CMD patient (n = 4) and control individual (n = 4) were run on the Gas chromatography–mass spectrometry GC–MS (Shimadzu GC–MS QP2010). Differentiated myotubes were first washed with isotonic sodium chloride (0.09% NaCl) solution quickly. The metabolism of cells was quenched by addition of 1 ml of methanol–water mix (9:1 v/v) and then the culture flasks were soaked into liquid nitrogen to freeze the cells. The frozen cells were scraped off into eppendorf tubes and culture flasks were washed with additional 1 ml of methanol: water mix (9:1, v/v), then 2 ml of cell extracts were centrifuged for 10 min at 15,000 rpm. Supernatant was transferred into a new tube and stored at -20 °C until analysis. Pellet was used for protein isolation and total protein concentrations were determined using Pierce BCA Protein Assay Kit (Thermo Fisher Scientific, Waltham, MA, USA). All samples were normalized based on the protein concentration before running on the instrument (3 mg protein/ 400 μl) and the cell extracts were evaporated to dryness in a vacuum dryer concentrator (Labconco Refrigerated CentriVap Vacuum Concentrator). GC–MS based metabolomic analysis were performed as described previously^61^. Detailed method parameters are given in the Supplementary information.

### Fluxomic analysis

Unlike metabolomic analysis, after washing the cells with isotonic NaCl solution quickly, 1 ml of growth medium containing 30% H_2_[^18^O] was added to culture flasks and incubated at 37 °C for 5 min to label phoshometabolites and Krebs Cycle intermediates. Subsequently, labeling medium was removed and cells were quickly washed with isotonic NaCl solution. Following washing step, all other stages were proceeded to be the same as for the untargeted metabolomic analysis. GC–MS based fluxomics analysis were performed as described previously^62,63^. The detailed parameters are given in the Supplementary Information.

### Quantification of phosphonucleotides

The cell extracts obtained from the metabolomic analysis were analysed using LC–MS/MS method for the quantification of 18 phosphonucleotides (AMP, ADP, ATP, GMP, GDP, GTP, TMP, TDP, TTP, IMP, IDP, ITP, CMP, CDP, CTP, UMP, UDP and UTP). The standards used in the calibration curves were prepared daily. The detailed method parameters are given in the Supplementary Information. Also, gradient elution of LC–MS/MS and the optimum MRM parameters for phosphonucleotides are shown in Supplementary Table S1 and S2, respectively.

### Data analysis

The data matrices obtained from GC–MS based metabolomic analysis were normalized according to desmin ratios. The missing values in the data was filled with the half-value of the smallest concentration in the metabolite group. Then, obtained data matrix was transferred to the SIMCA-P+ (version 13.0); partial least squares differentiation analysis (PLS-DA) were created to determine outliers and differences between control and patient groups (SI). Finally, pathway analysis was performed on significantly variable features obtained from metabolomic, and fluxomic analysis using Metaboanalyst 4.0.

### Statistical analysis

Differences between data sets were evaluated statistically by using the Mann–Whitney *U*-test (data were presented as median, minimum and maximum) or Student’s *t*-test (data were presented as mean ± standard deviation). Statistical analyses were performed using the GraphPad Prism 5.0 software (GraphPad Software, Inc., San Diego, CA). The statistically significance was accepted as *p* < 0.05.

## Supplementary Information

Supplementary Information.
