# Supplementary material for: Reduced mitochondrial fission and impaired energy metabolism in human primary skeletal muscle cells of Megaconial Congenital Muscular Dystrophy
Source: Sci Rep. 2021 Sep 13;11:18161. doi: 10.1038/s41598-021-97294-4 (PMC8438035; doi:10.1038/s41598-021-97294-4)
Supplement: Supplementary file 1 — Supplementary Information. [file 41598_2021_97294_MOESM1_ESM.pdf]

Reduced mitochondrial fission and impaired energy metabolism in human primary skeletal muscle cells of Megaconial Congenital Muscular Dystrophy

Evrim Aksu Menges, Cemil Can Eylem, Emirhan Nemutlu, Merve Gizer, Petek Korkusuz, Haluk Topaloglu, Beril Talim, Burcu Balci-Hayta

SUPPLEMENTARY INFORMATION

I. Metabolomics analysis

- **Derivatization:** Residuals were methoxyaminated using 20 µl of methoxamine hydrochloride in pyridine (20 mg/1 ml) for 90 minutes at 30°C and derivatized using 80 µL of N-methyl-N- (trimethylsilyl) trifluoroacetamide (MSTFA) at 37°C for 30 minutes. The samples were transferred into GC-MS vials.
- **Instrument parameters:** The primary myoblast cells of megaconial CMD patients (n=4) and control individual (n = 4) cell extracts were run on the GC-MS (Shimadzu GCMS-QP2010 Ultra) with DB-5MS stationary phase column (30 m +10 m duraguard × 0.25 mm i.d. and 0.25-µm film thickness). 1 µl sample was injected to the column with splitless mode. The run time was set 37.5 min using oven temperature program. The parameters of GC-MS as follows: MSD transition temperature was at 290°C with a flow rate of 0.99 ml/min and solvent delay time was 5.90 minutes. The complex chromatograms were deconvoluted using AMDIS and the retention time correction and data matrixes creation were done using SpectConnect software.

II. Fluxomics analysis

- **Derivatization:** The labeled samples (3 mg protein/ 400µl) (n=8) was evaporated to dryness in a vacuum dryer concentrator and derivatized with MSTFA with 1% TMCS and analysed as in metabolomics analysis. Once analysis completed, the labeling percentages of metabolites via ^{18}O were calculated using the peak areas of each labeled isotopes. These values were used to calculate the total labeling percentage of ^{18}O by the following formula.

$$\text{Total } ^{18}\text{O labeling } \% = \left(\sum_{i=1}^{n} i \; x \; ^{18}\text{O}_i\% \right) / \; n \; x \; \text{H}_2[^{18}\text{O}]\%$$

n: Total labeled oxygen in molecule

i: Number of labeled oxygens with $H_2[^{18}O]$

III. Quantification of phosphonucleotides

The quantification of 18 phosphonucleotides (AMP, ADP, ATP, GMP, GDP, GTP, TMP, TDP, TTP, IMP, IDP, ITP, CMP, CDP, CTP, UMP, UDP and UTP) were performed using LC-MS/MS (Shimadzu 8030). The stock solution of each phosphonucleotide (5 mM) were prepared in methanol:water (50:50, v/v). Then, the standard solution of calibration curves (0.1, 0.05, 0.01, 0.005, 0.001, 0.0005 and 0.0001 mM) were prepared daily from the stock solutions of phosphonucleotides.

- **Instrument Parameters:** The analyses were performed with an LC–MS/MS (Shimadzu LC-20AXR system coupled to a triple quadrupole tandem mass spectrometer Shimadzu 8030) using a Merck ZIC-pHILIC (150 mm x 2.1 mm, 5 µm) column with a 0.17 ml/min flow rate constituted from 20 mM ammonium carbonate containing 5% acetonitrile (A) and acetonitrile containing 5% water (B) in gradient elution mode as Supplement Table 1. The column temperature was set 30°C. The sample injection volume and run time was set 10 µl and 30 min, respectively. The ESI-MS/MS conditions were as follows: interface voltage, 4.5 kV; nebulizer gas flow rate, 3 ml/min; drying gas flow rate, 15 L/min; desolvation line temperature, 250 °C; heat block temperature, 400 °C. Negative ionization mode was used in multiple reaction monitoring (MRM) for the quantification of each oligophospho nucleotide. The MRM parameters was set as in the Supplement Table 2.

Supplementary Table 1. Gradient elution of LC-MS/MS

Time (min)	Pump B
**1.00**	95
**15.00**	50
**21.00**	5
**25.00**	5
**26.00**	95
**30.00**	Stop

Supplementary Table 2. The optimum MRM parameters for phosphonucleotides

	Precursor Ion	Fragment Ion	Dwell Time	Q1*	CE **	Q3***
**ATP**	506.00	158.90	100.0	20.0	32.0	28.0
**ADP**	426.00	158.90	100.0	12.0	28.0	30.0
**AMP**	346.10	133.80	100.0	12.0	32.0	20.0
**GMP**	362.10	149.90	100.0	14.0	35.0	12.0
**GDP**	442.00	158.90	100.0	17.0	29.0	13.0
**GTP**	522.00	158.90	100.0	20.0	32.0	27.0
**CMP**	322.00	138.90	100.0	12.0	25.0	25.0
**CDP**	402.00	158.80	100.0	15.0	26.0	30.0
**CTP**	482.00	158.90	100.0	11.0	29.0	26.0
**TMP**	321.10	194.70	100.0	12.0	19.0	17.0
**TDP**	401.00	158.90	100.0	15.0	27.0	30.0
**TTP**	481.00	158.80	100.0	11.0	29.0	30.0
**UMP**	323.00	111.00	100.0	12.0	30.0	21.0
**UDP**	403.00	158.80	100.0	15.0	28.0	27.0
**UTP**	483.00	158.90	100.0	14.0	33.0	28.0
**IMP**	347.10	134.90	100.0	13.0	35.0	20.0
**IDP**	427.00	158.90	100.0	16.0	29.0	29.0
**ITP**	507.00	158.90	100.0	20.0	35.0	28.0

*Quadrupole 1 pre bias voltage, **CE: Collision energy and *** Quadrupole 3 pre bias voltage

SUPPLEMENTARY FIGURES

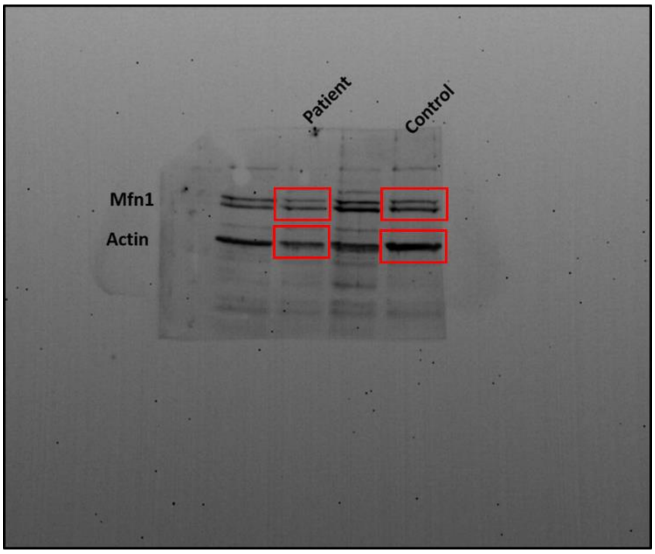

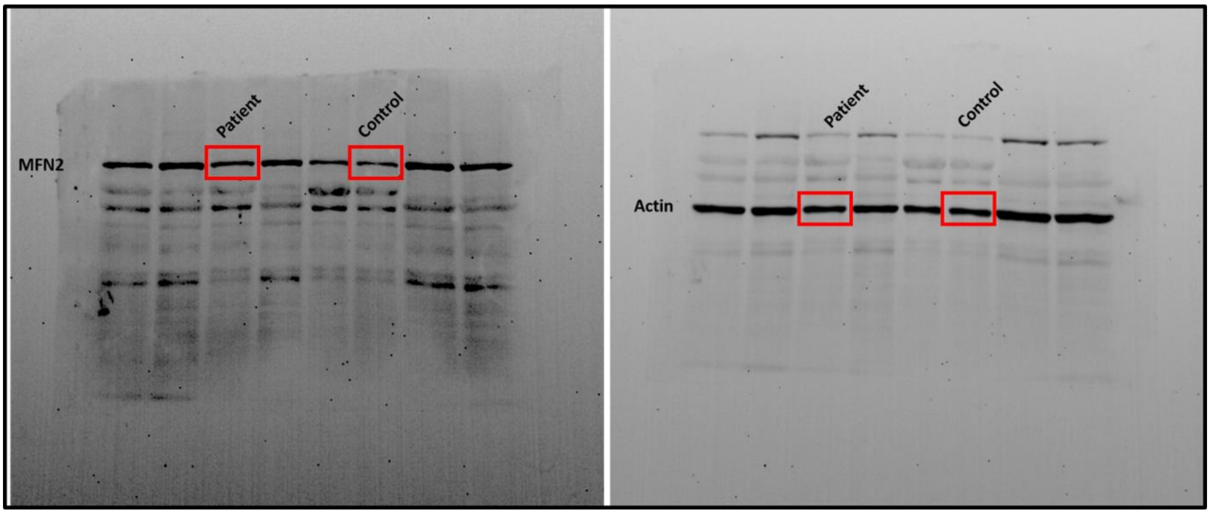

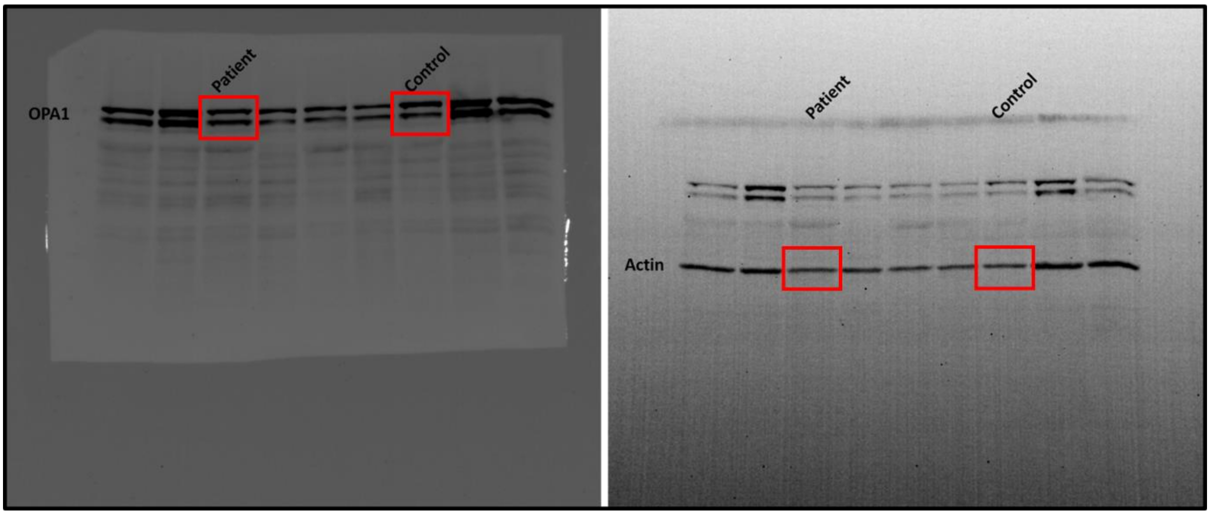

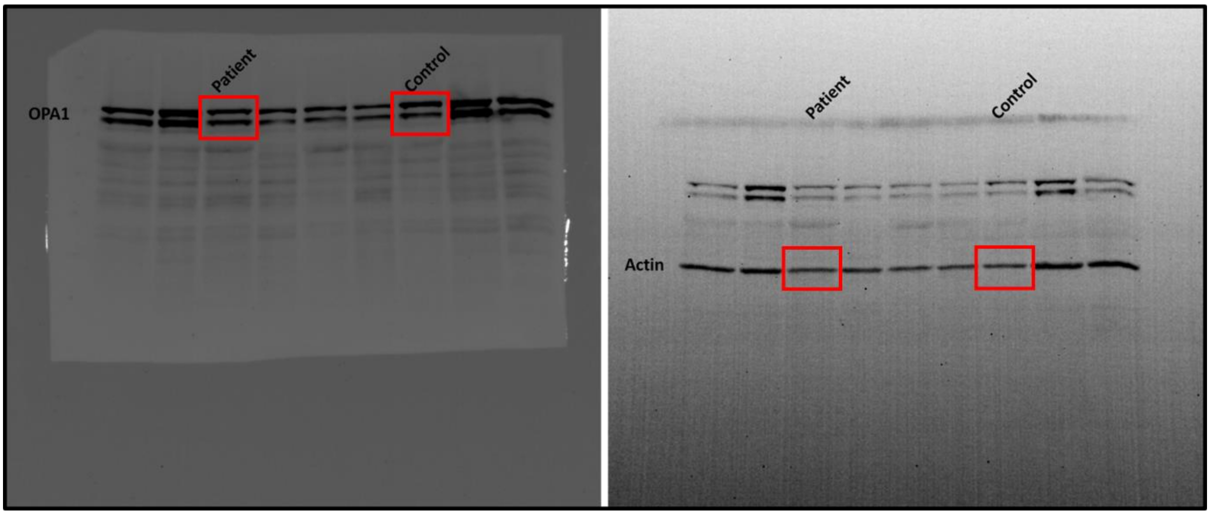
OPA1
Patient
Control
Patient
Control
Actin

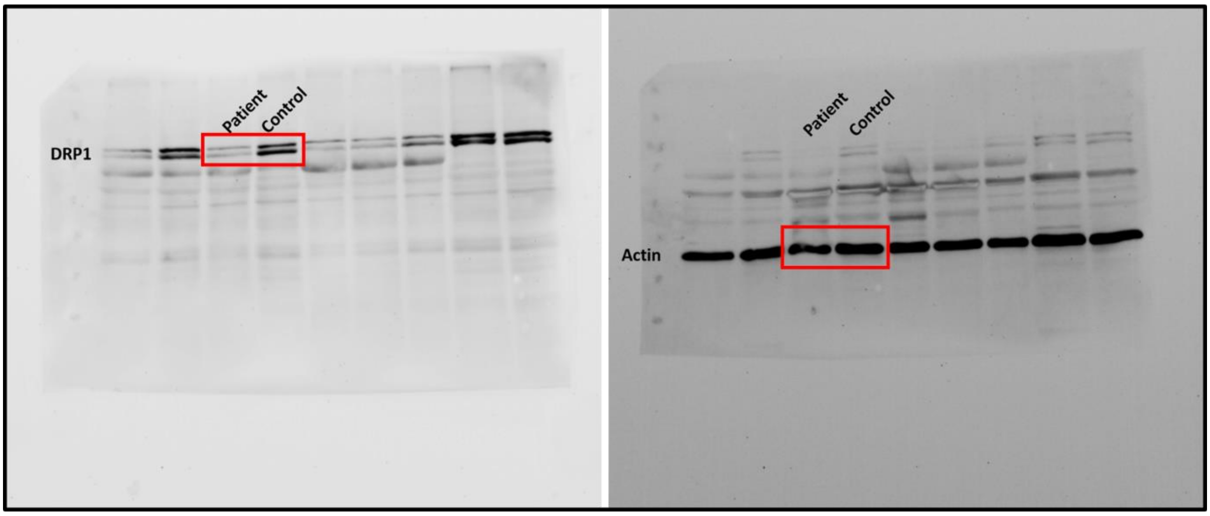
DRP1
Patient
Control
Patient
Control
Actin

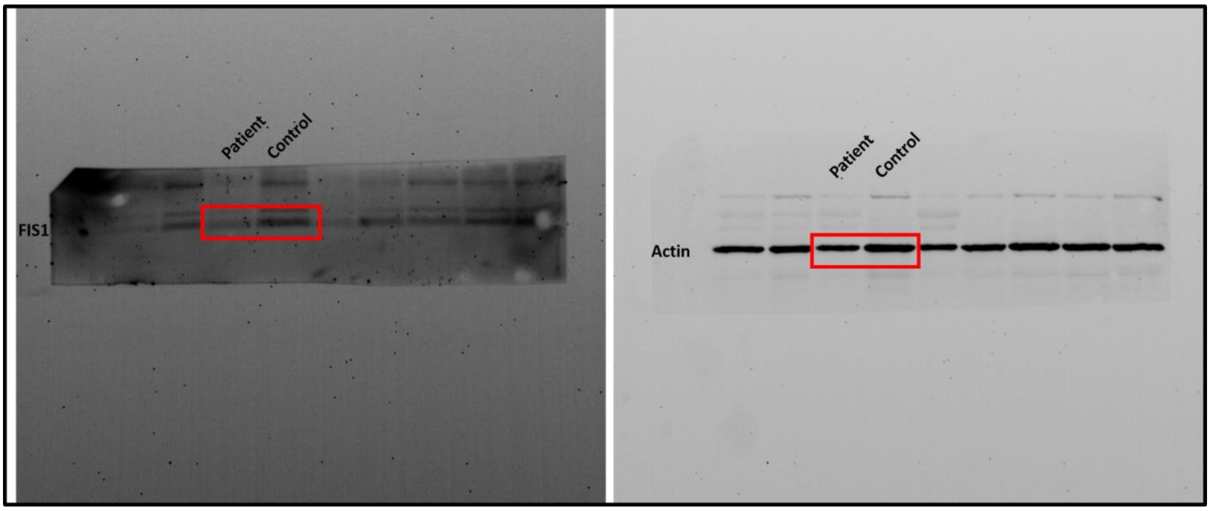
FIS1
Patient
Control
Patient
Control
Actin

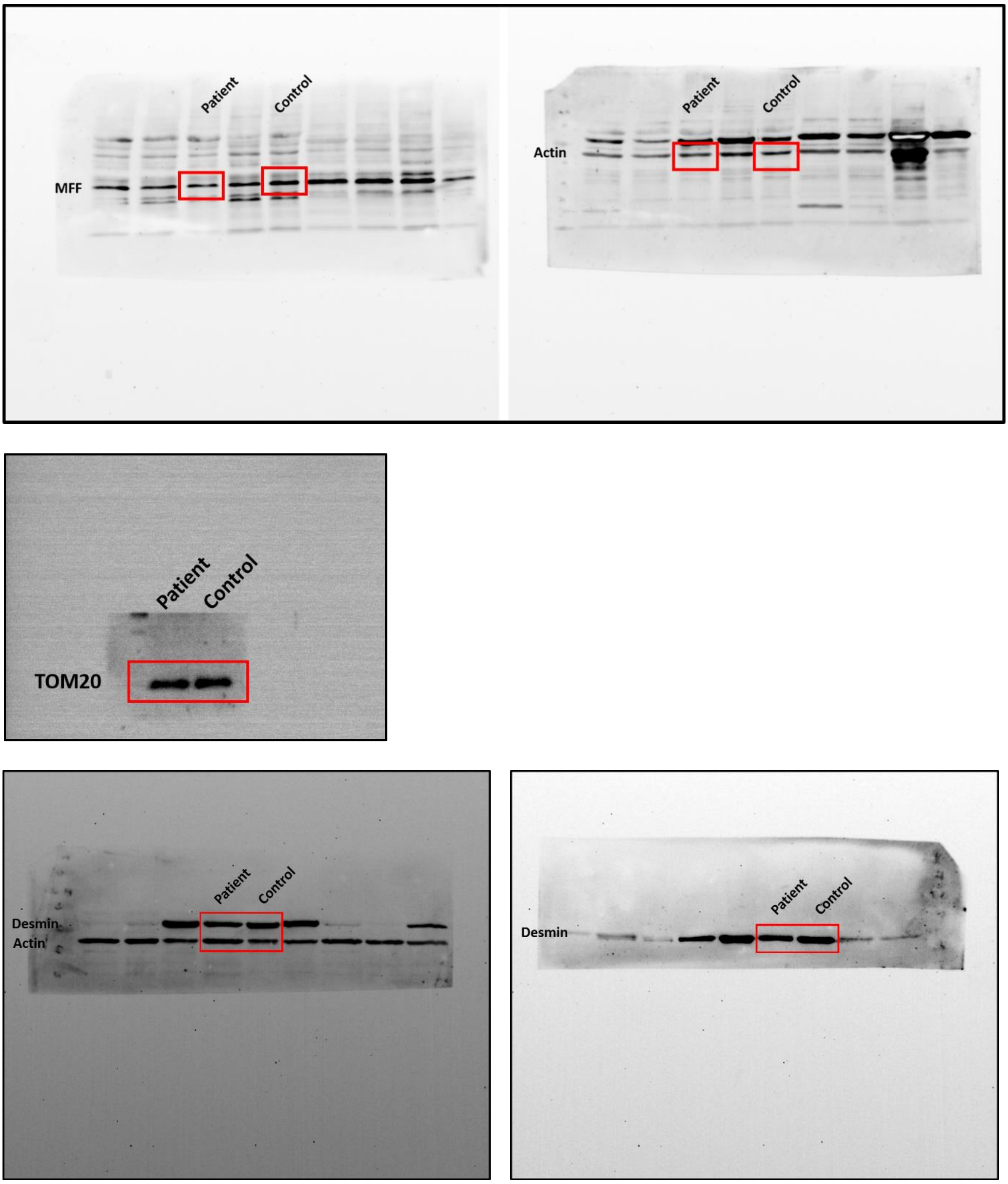

Supplementary figure S1. Uncropped images of Western blotting**.** Related bands are denoted by red boxes. Lanes other than patient and control are unrelated samples.

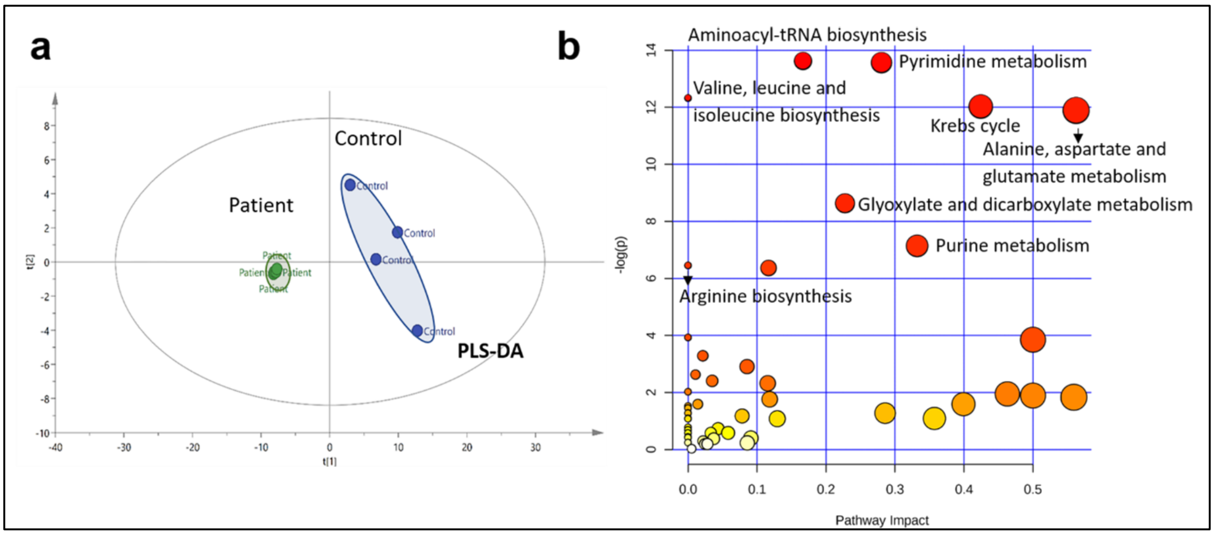

Supplementary Figure S2. PLS-DA scattering plots (R^2X: 0.905, R^2Y: 0.978, Q^2:0.875) (A), and Pathway impact graphs (B) obtained from primary skeletal muscle cells of the Megaconial CMD patient and the control.

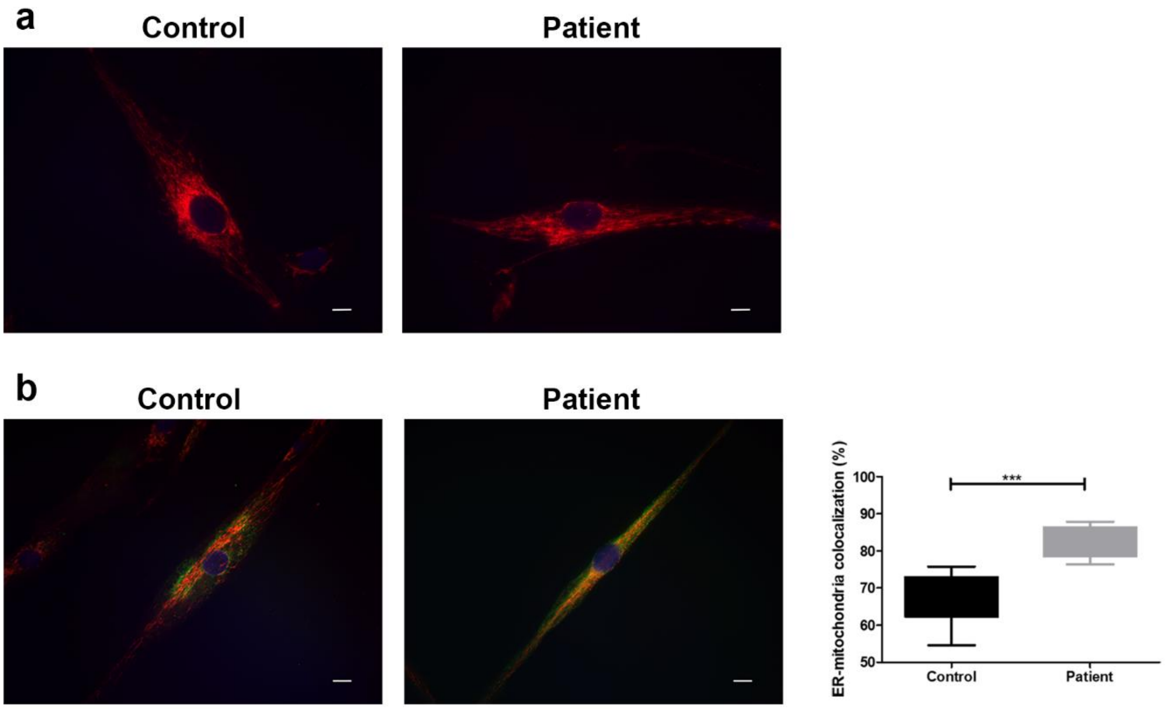

Supplementary Figure S3. Immunofluorescence analysis of ER (A) The morphology of ER in primary skeletal muscle cells of the Megaconial CMD patient and control. Red/Texas Red: Calnexin/ER; Blue: DAPI/nuclei. (B) Colocalization analysis of mitochondria and ER in primary skeletal muscle cells of the Megaconial CMD patient and control. Green/FITC: PDI/ER; Red/Texas Red: Tom20/mitochondria; Blue: DAPI/nuclei. Scale bars: 10μm. Quantifications were performed on 30 primary skeletal muscle cells in both patient and control samples.
